# The *El Valor de Nuestra Salud* clustered randomized controlled trial store-based intervention to promote fruit and vegetable purchasing and consumption

**DOI:** 10.1186/s12966-021-01220-w

**Published:** 2022-02-17

**Authors:** Guadalupe X. Ayala, Julie L. Pickrel, Barbara Baquero, Jennifer Sanchez-Flack, Shih-Fan Lin, George Belch, Cheryl L. Rock, Laura Linnan, Joel Gittelsohn, Ming Ji, John P. Elder, Joni Mayer

**Affiliations:** 1 Division of Health Promotion and Behavioral Sciences, School of Public Health, San Diego State University, San Diego, USA; 2 Institute for Behavioral and Community Health, San Diego State University Research Foundation, 9245 Sky Park Court, Suite 220, San Diego, CA 92123 USA; 3 Health System and Population Health, School of Public Health, University of Washington, Box 35480, Seattle, WA 98195 USA; 4 Department of Pediatrics, University of Illinois Cancer Center, Chicago, USA; 5 Institute for Health Research and Policy, University of Illinois at Chicago, 1747 West Roosevelt Road, WROB 478, Chicago, IL 60608 USA; 6 Marketing Department, Fowler College of Business, San Diego State University, 5500 Campanile Drive, San Diego, CA 92182 USA; 7 Department of Family Medicine, School of Medicine, University of California, San Diego, 9500 Gilman Drive #0901, La Jolla, CA 92093 USA; 8 Department of Health Behavior, Gillings School of Global Public Health, University of North Carolina at Chapel Hill, 359 Rosenau Hall, 135 Dauer Drive, Campus Box 7440, Chapel Hill, NC 27599 USA; 9 Department of International Health, Center for Human Nutrition, Johns Hopkins Bloomberg School of Public Health, 615 North Wolfe St, Baltimore, MD 21205-2179 USA; 10 College of Nursing, University of South Florida, Tampa, FL 12901 USA

**Keywords:** Food environment, Food store, Food purchasing, Latinos/Hispanics, Fruits, Vegetables

## Abstract

**Background:**

Modifying the environment to promote healthy foods is a population-based approach for improving diet. This study evaluated the outcome effectiveness of a food store intervention that used structural and social change strategies to promote fruits and vegetables. It was hypothesized that intervention versus control store customers would improve their consumption of fruits and vegetables at 6 months.

**Trial design:**

Clustered randomized controlled trial

**Methods:**

Sixteen pair-matched stores were randomized to an intervention or wait-list control condition. With the research team’s support, intervention stores modified the availability, accessibility, and promotion of fruits and vegetables, including augmenting produce displays within the store and building employees’ capacity to place and promote fruits and vegetables throughout the store (Phase 1), followed by the delivery of a customer-directed marketing campaign for 6 months (Phase 2). From months 7 to 12, stores were encouraged to maintain strategies on their own (Phase 3). Customer-reported daily fruit and vegetable consumption (cups/day) were collected by blinded research assistants at three time-points (baseline, 6 months and 12 months post-baseline) from 369 participating customers (an average of 23/store). Secondary outcomes included customer-reported fruit and vegetable purchasing and other behaviors.

**Results:**

The study retained the 16 stores and most customers at 6 (91%) and 12 (89%) months. Although significant differences were not observed in the overall sample for vegetable consumption, male customers of intervention versus control stores consumed significantly more fruit daily at 6 months [mean (standard deviation) cups at baseline and six months; intervention: 1.6 (1.5) to 1.6 (1.5) vs. control: 1.4 (1.2) to 1.1 (0.8)]. However, this difference was not observed at 12 months, or among females. There was an overall increase in dollars spent at the targeted store in the intervention versus control condition among male versus female customers at 6 months; however, no change was observed in the percent of dollars spent on fruits and vegetables at the targeted store. Frequency of shopping at the targeted store did not modify intervention effects.

**Conclusions:**

Structural and social change interventions can modify customers’ behavior in the short-term. Future research should consider methods for achieving longer-term changes, and potential generalizability to other products (e.g., energy-dense sweet and savory products).

**Trial registration:**

NCT01475526

**Supplementary Information:**

The online version contains supplementary material available at 10.1186/s12966-021-01220-w.

## Background

Fruit and vegetable consumption is associated with the prevention of various chronic health conditions [1–3] and weight gain [4] among adults. Despite efforts to promote consumption, few United States (US) residents meet 2015-2020 Dietary Guidelines for fruits (12.2% consume 1-2 cups per day) and vegetables (9.3% consume 2-3 cups per day) [5]. Women are more likely to meet recommendations than men for both fruits (15.1 vs. 9.2%, respectively) and vegetables (10.9 vs.7.6%, respectively). Latinos/Hispanics, in particular, exhibit gender disparities in fruit and vegetable consumption, with more Latinas consuming fruits and vegetables five or more times per day compared with Latino men (28.3 vs. 19.8%, respectively) [6]. Thus, strategies are needed to increase consumption of fruits and vegetables among Latino/Hispanic adults, and men in particular, to help prevent and control obesity and other diet-related health conditions. This is also relevant to U.S. immigrants given the shift to a less healthy dietary profile the longer they live in the US (e.g., consuming fewer fruits and vegetables [7]). These changes are driven, in part, by changes in food purchasing behaviors [8].

There is ample evidence that the food environment determines what we eat [9, 10]. The process by which this occurs includes having access to healthy options within a community and within a store [11, 12]. Community nutrition environment studies show that neighborhoods with fewer supermarkets [13] and more supercenters and convenience stores [14] have higher obesity rates; however, these findings are not always consistent [15]. Likewise, products made available and promoted within the store (the consumer nutrition environment) determine what is purchased, particularly given evidence that a majority of purchasing decisions are made in the store [16]. Food purchasing is ultimately associated with what is consumed, including for fruits and vegetables [17], which may explain why stores having a higher ratio of healthy versus less healthy foods (i.e., low nutrient-dense) on store shelves was associated with a lower risk of overweight and obesity among residents [15]. Testing whether a store-based intervention increases the purchasing and consumption of fruits and vegetables is a natural extension of this work.

Interventions to improve the consumer nutrition environment within small food stores [18] and grocery stores/supermarkets [19] have shown mixed results, and there is limited evidence from rigorously controlled studies with data collected from a cohort of customers. A 2016 systematic review determined that consumer nutrition environment interventions within food stores are effective at increasing the purchase of targeted products, particularly when a combination of strategies is used to increase the availability and promotion of these products [20]. However, except for virtually-delivered interventions [21], studies do not commonly follow a cohort of customers to examine for intervention effects on their purchasing and consumption of targeted products. We refined a previous efficacious [22] and effectively implemented [23] intervention showing improvements in fruit and vegetable consumption among a small cohort of customers in four stores located in North Carolina, USA.

This study [24], conducted in 16 stores in San Diego County, CA along the USA-Mexico border, had as its objectives to determine whether an in-store intervention involving structural and social changes would impact customer-reported fruit and vegetable purchasing and consumption (primary outcomes), and other dietary behaviors (e.g., variety of fruits and vegetables consumed, dietary behavioral strategies to promote fruit and vegetable consumption, and percent energy from fat) at 6 months post-baseline compared with customers in a wait-list control condition. During months 1 through 2 (Phase 1), the intervention was delivered with support from the research team, including providing resources to augment the store’s produce displays and capacity-building to store employees and managers. Months 3 through 6 (Phase 2) involved the addition of customer-directed activities. During months 7 to 12 (Phase 3), the intervention stores were responsible for maintaining strategies on their own. It was hypothesized that intervention versus control customers would report higher fruit and vegetable consumption, as well as more dollars spent on fruits and vegetables and improved dietary behaviors at the 6-months post-baseline evaluation time-point. The 12-month evaluation time-point examined what happened with customer consumption and purchasing of fruits and vegetables when the stores were solely responsible for intervention implementation activities for six months. Finally, we explored whether differential intervention effects were observed by customer gender to determine the potential for using these types of intervention strategies to reduce gender-based disparities in healthy behaviors [25], notwithstanding recent findings showing few gender differences in indicators of diet quality from foods purchased [26].

## Methods

### Study design

*El Valor de Nuestra Salud* (The Value of our Health; *El Valor*) was a clustered randomized controlled trial (C-RCT) with 16 pair-matched limited assortment Latino/Hispanic-focused food stores located in an urban community; stores were randomized to an intervention versus wait-list control condition. The store-based intervention sought to promote fruit and vegetable purchasing, consumption, and other dietary behaviors among customers through structural and social changes in the stores and involving store managers and employees. Stores and customers were recruited in waves; store and customer baseline data were collected at the same time for pair-matched stores and occurred between October, 2011 and October, 2013. Customer primary outcome data, the focus of this paper, were collected 6 and 12 months later. All study protocols were approved by the Institutional Review Board of San Diego State University and memorandums of understanding were established with all stores. Details on all aspects of the study are published in the protocol paper [24].

### Setting

There were three important characteristics of the stores: limited assortment, independently-owned, and Latino/Hispanic-focused. Limited assortment food stores have fewer product categories than traditional supermarkets but more than convenience stores [27]. Their focus is on meeting daily consumable needs, including fresh perishables [28]. They share similarities to fruit and vegetable stores in New Zealand [29], in their equal or better availability of healthy products (e.g., lower cost) compared with supermarkets [30]. In addition, given a greater number of service departments in limited assortment food stores compared with convenience stores, they offer more ways in which to promote fruit and vegetable purchasing. Although the overall sales of independently-owned stores are low, they serve an important role in ensuring access to fresh consumables in rural and low-income communities [31]. For intervention purposes, they are an ideal partner given their ability to make decision autonomously; problems can often be solved immediately to ensure rigorous implementation of an RCT [23]. As introduced in the Background, the focus of this intervention trial was on reaching the US Latino/Hispanic (predominantly Mexican-origin) population given observed health disparities, by working with Latino/Hispanic-focused stores. These stores, referred to as ‘*tiendas’*, have been identified as an important source of foods and beverages purchased in both San Diego, California [8], and Burlington, North Carolina, USA [32]. Store managers and employees of *tiendas* have an appreciation of what might influence customer purchasing behavior [33].

Stores were located throughout San Diego County, California, USA, including in the mid-city and North San Diego County regions. San Diego County has a large Mexican-origin population. During the baseline data collection period, 32% of adult residents in San Diego County identified as Latino/Hispanic, 37% reported speaking a language other than English including 66% of whom reported Spanish as their dominant language, and 10% were living in poverty [34].

### Store, manager, and customer recruitment

As noted in the protocol paper [24], stores were enumerated from various sources and screened for eligibility. Eligibility criteria included: being an independently-owned limited assortment food store with a largely Latino/Hispanic customer-base and one that catered to this customer base through the brands offered and marketing language used (Spanish language or Spanish and English). Store employees were bilingual or Spanish-language dominant. Eligible stores had to carry fresh produce, have a serviced meat department, and be located in a neighborhood with at least 20% Latino/Hispanic residents. Unlike some small store interventions with Latinos/Hispanics [35], we sought to work with limited assortment food stores with some infrastructure and product already available but inadequate and/or insufficient to effectively promote the sale of fruits and vegetables in their various forms. At the other extreme, however, stores classified in the North American Industry Classification System® as supermarkets were not eligible given their already-available infrastructure for merchandising fresh produce and often inability to make independent decisions.

Eligibility criteria were primarily assessed during an in-person visit to the store. Once recruited, stores were pair-matched at baseline to balance potential differences across condition (e.g., store size, availability of a prepared food department), and to minimize the potential for cross contamination across conditions by ensuring stores were at least one mile apart. After baseline data collection was completed, pair-matched stores were randomized to the intervention or wait-list control condition.

**Fig. 1:**
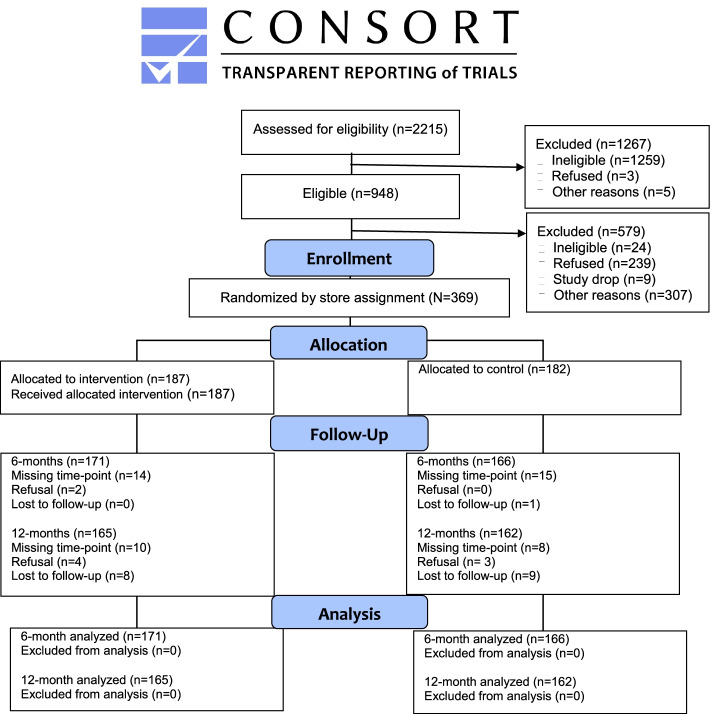
CONSORT flow diagram

Approximately 23 eligible customers self-identifying as Latino/Hispanic were recruited for the evaluation cohort per store. Cohort inclusion criteria were: at least 18 years of age; able to read Spanish; among the primary household grocery shoppers; shopped at the targeted store at least once per week to maximize intervention exposure (if applicable); did not shop at other study stores at least once a month or more to minimize potential cross contamination; purchased food and beverage products at the targeted store; no dietary restrictions; reported consuming no more than 4 cups/day of fruits and vegetables [36]; having a telephone at which the individual could be reached; not planning to leave the study area during the study period; and not participating in any other study to promote healthy eating. Only one individual per household was allowed to participate to minimize additional sources of clustering. A time sampling approach was used to recruit customers to minimize selection bias. If the customer was eligible and agreed to participate, the research assistant administered the baseline data collection protocol immediately or scheduled a future visit to collect these data. If the customer refused during recruitment or after, the refusal was noted. If the customer was ineligible, the customer was thanked and the ineligibility criteria were noted. Customer recruitment took between four and 13 weeks per store, with the number of recruitment visits to stores ranging from six to 21*.* Our CONSORT figure (see Fig. 1) provides information on recruitment yield and retention rates (see Additional file 1 for our CONSORT checklist).

### Intervention condition

**Fig. 2:**
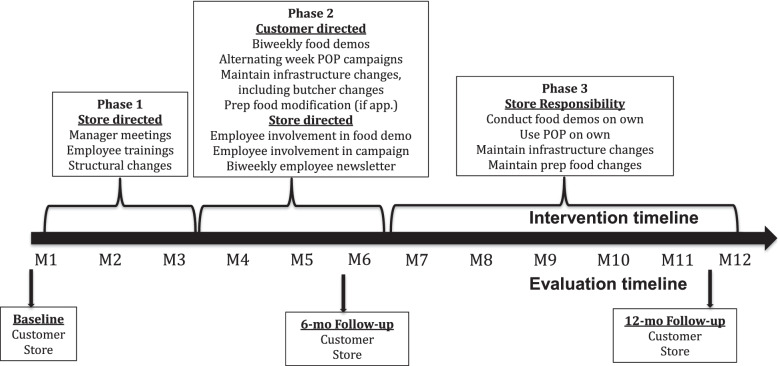
Timeline of intervention and evaluation activities

**Fig. 3:**
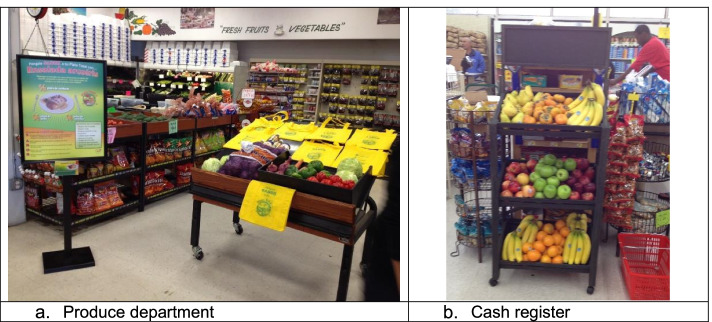
Intervention implementation. **a** Produce department. **b** Cash register

Figure 2 depicts the three intervention phases and the evaluation time-points. Figure 3 shows photographs of intervention implementation in stores, in the produce department and near a cash register. The change strategies were informed by the Socio-Ecologic Framework which acknowledges the multiple sources of influence on fruit and vegetable purchasing [37], as well as formative research conducted with store managers and employees [33] and store audits conducted in similar stores [30]. Social Marketing Theory [38] and McGuire’s Input-Output Matrix [39] informed the design of the marketing campaign, including identifying important placement and promotion considerations.

Briefly, for months 1 through 2, Phase 1, structural and social change strategies were directed at each store individually via in-person meetings with the manager and group trainings with employees, both conducted by the research intervention coordinator. Individual meetings were used to identify feasible and potentially sustainable approaches relevant to each store. Structural changes were facilitated through the provision of $2000 to purchase equipment and other infrastructure to display and promote fruits and vegetables (e.g., cold bars to display ready-to-eat fresh-cut fruits and vegetables) in the produce department and elsewhere (e.g., near cash registers; see Fig. 3), as well as to facilitate changes to the serviced meat (e.g., offer ready-to-cook fajitas) and prepared foods departments (if applicable; e.g., add vegetables to an existing prepared foods dish; offer a side vegetable option). Facilitated by the manager and lead butcher, stores were asked to select a minimum of two of three changes to be implemented within the serviced meat department to cross promote or cross merchandise fresh fruits and vegetables with fresh meat offerings. Similar strategies were used with stores having a prepared foods department, however, changes were limited to a minimum of one of three changes.

Social changes were accomplished through four one-hour employee trainings delivered in groups by the research intervention coordinator and addressing the topics of customer service, fruit and vegetable product knowledge, merchandising, and implementing an integrated campaign. A minimum of 25% of employees were targeted to receive the full training, and a 10-minute training was introduced after implementation in the first intervention store to serve as an introduction to the project for employees who did not participate in the full training. The manager installed posters in the employee areas of the stores reinforcing the training messages (e.g., how to provide excellent customer service).

During months 3 through 6, Phase 2 (see Fig. 2), structural and social change strategies in the store were mostly directed toward customers, with reinforcement strategies directed to employees. Phase 2 involved the implementation of an in-store marketing campaign, similar to nudging strategies [40]. The campaign included the installation of point-of-purchase (POP) marketing materials throughout the store to promote fruits and vegetables and the delivery of nine biweekly food demonstrations featuring recipes and tastings that highlighted a variety of fruits and vegetables. POP marketing materials included shelf danglers, aisle violators, and posters. Select POP marketing materials rotated every two weeks through a series of nine recipes that were featured at food demonstrations. At the food demonstrations, recipe cards, reusable grocery bags, and magnetic calendars were distributed along with food samples, allowing customers to taste potentially new fruits and vegetables and/or fruits and vegetables prepared in a different way. Food demonstrations were identified by store managers and employees as an important strategy for reaching customers [33]. In the marketing materials and during the food demonstrations, key behavioral messages were conveyed through the use of the *Plato Total* (Total Plate; similar to the USDA My Plate guidelines) image and the words *SABOR* (Taste; acronym stands for five dietary behaviors: S=sustituir/substitution, A=añadir/addition, B=balancear/balance, O=optar por variedad/opt for variety, and R=reformar/reform [preparation methods]) on materials and talking points. All customer-directed materials, including all POP marketing materials, were in Spanish. To maximize exposure to the intervention among participants in the intervention condition, an introductory letter, a reusable grocery bag imprinted with the Plato Total image and SABOR messages, and a magnet calendar were mailed to their homes. This was followed by bi-weekly mailings of the recipe “postcards” announcing the next scheduled food demonstrations.

During Phase 2, employees received newsletters consistent with each new food demonstration; the newsletter included reinforcing messages from the training related to customer service and how to promote the upcoming campaign, including specific talking points to share with customers, in English and Spanish. These newsletters were delivered directly to the employees or left with the manager for distribution. Managers were encouraged to appoint an employee to assist with the food demonstration and campaign installation to build their skills for Phase 3.

During months 7 through 12, Phase 3, the research intervention coordinator conducted in-person visits with store managers to encourage them to continue maintaining the structural changes, food demonstrations, and POP campaign implemented in all store departments (e.g., produce, service meat, prepared foods) and other locations in the stores. Stores were provided with POP campaign materials to use as they felt most appropriate.

In terms of intervention implementation, there were few variations in the implementation of the intervention from what was originally planned [24, 41], and no differences by store. Overall, employee trainings took more time than anticipated given the need to schedule smaller groups of employees at any one time and structural changes cost more than originally anticipated causing slight modifications to recommendations (see Additional file 2 for our TIDieR checklist) [41]. Using data gathered to examine changes in the observed environment, Sanchez-Flack et al. [42] found evidence for intervention fidelity of the promotional campaign (e.g., more fruit and vegetable promotions overall and outside the produce department in intervention versus control stores at 6-months post baseline). However, no intervention effects were found for observed availability of fruits and vegetables and shelf space devoted to it, suggesting that the environment may not have sufficiently changed to impact purchasing.

### Control condition

Stores assigned to the wait-list control condition engaged in evaluation activities only. Control stores were offered the intervention training materials and technical assistance following completion of the 12-month evaluation time-point. Funding limits prevented us from offering the stores the opportunity to purchase equipment and conduct the biweekly food demonstrations. Four (50%) of the control stores took advantage of the trainings and materials offered.

### Outcome evaluation protocol and measures

Our outcome evaluation protocol included obtaining data from customers and managers through in-person structured interviews and store data through in-person store audits. The research assistants who conducted the interviews were predominantly bilingual, and in some cases bicultural, young adult males and females. They received training in data collection and human subjects’ research, including completion of human subjects training. Consistent with previous study protocols [22], research assistants who conducted the store audits were trained through extensive practice to capture detailed information about the store environment. Inter-rater reliability was established prior to involvement in formal store audit data collection.

#### Customer interviews

Customer data collection protocols occurred at baseline and were repeated 6 and 12 months later, with interviews and weight measurements (weight not reported here) occurring in their homes or a moderately private location in the community (e.g., in or outside the store where possible, nearby park or recreation center). In-person data collection was emphasized but telephone interview data collection was permitted for several cases at the 6- and 12-month follow-up periods. During follow-up periods, research assistants were blinded to customer condition assignment by not scheduling the follow-up data collection visits with customers at the stores.

Customers were interviewed about their dietary intake, purchasing behaviors, other dietary behaviors (past month variety of fruits and vegetables, dietary behavioral strategies to increase fruit and vegetable consumption, and percent energy from fat), as well as their socio-demographic characteristics. *Daily cups of fruits and daily cups of vegetables* were assessed using the National Cancer Institute (NCI) All-Day fruit and vegetable screener which asks about 10 sources of fruits and vegetables and how frequently (i.e., never to five or more times per day) customers consumed each source during the past month [43, 44]. Each questions on frequency was followed by a question on quantity consumed with appropriate quantities specified for each source (e.g., fruit: less than one medium fruit/less than ½ cup to more than 2 medium fruits/more than 1 cup; vegetable soup: less than 1 cup/less than 1 full bowl to more than 3 cups/more than 3 full bowls). Food models and other similar tools were used to estimate portion sizes and a calendar was used to assist with weekly and monthly recall [45]. Using NCI scoring protocols [46], two scores were computed: daily cups of fruit (including the consumption of fruit and juice) and daily cups of vegetables (including the consumption of lettuce salad, other vegetables [e.g., stews, stir-fry], tomato sauce, and vegetable soups).

*Purchasing* was assessed with five questions on self-reported shopping frequency and dollars spent during a typical week for groceries overall, for fruits and vegetables, and specifically within the targeted store. Questions were derived from previous studies conducted with the target population, thus ensuring their relevance to them [8]; however, they were not considered proxy clinical indicators of nutrient intakes [17]. Frequency of shopping for fruits and vegetables was assessed with one question asking how often the customers shopped for fruits and vegetables from less than once per month to every day. Given the distribution of responses observed, the original six responses were recoded into three ordinal categories; 1=less than 1-2 times/week, 2=1-2 times/week, and 3=more than 1-2 times/week. Two questions asked about the weekly dollars spent on groceries overall and for fruits and vegetables specifically. These were followed by two questions that assessed how much of their grocery dollars and produce dollars were spent in the targeted store. The latter two questions allowed us to create an additional score representing the percent of weekly dollars spent on fruits and vegetables in the targeted store from among the total weekly dollars spent on groceries at the targeted store.

*Past month’s variety of fruits and variety of vegetables* were assessed with two items [22]. Each item, one listing 31 fruits and one listing 38 vegetables, asked the individual to indicate whether he/she had eaten each variety of fruit or vegetable in the past month. Consumption may have been in any form (raw or cooked) and from any source (home, restaurant, work, etc.). Variety confers health benefits with individuals characterized as having high variety fruit and vegetable consumption patterns being less at risk for being obese [47]. All varieties were summed to yield final scores of total number of varieties of fruits and total number of varieties of vegetables consumed in the past month.

*Dietary behavioral strategies* were assessed with three subscales using a modified version of an instrument that assessed behaviors associated with improvements in dietary intake [48–51]. Subscale scores for customers who missed more than 20% of the items in a subscale were not computed. One subscale assessed substitution behaviors (e.g., buy fruits for your snacks instead of other sweet snacks; five items; α=0.79). The second subscale assessed preparation behaviors that often begin during shopping (e.g., add vegetables, or more vegetables, to a dish you usually prepare; five items; α=0.68). The last subscale assessed behaviors associated with opting for a variety of products (e.g., use more kinds of vegetables than a dish calls for to add variety; six items; α=0.83). Responses are made on a frequency scale from 1=never/rarely to 4=usually and a mean score was computed for all three subscales with higher scores indicating more frequent use of the behavioral strategy.

*Percent energy from fat* was assessed with the NCI Fat screener [52]. Customers were read a list of foods and beverages and asked how often they consumed each during the past year from never to two or more times per day. Consistent with NCI protocols, reported frequency was recoded into daily consumption and then multiplied by the corresponding age- and gender-specific median serving sizes and the gender-specific estimated regression coefficients. Percent energy from fat was calculated from the sum of these food and beverage items.

Demographic information was obtained from the customers, to characterize the sample and to control for important sources of variance including people living in poverty, homeownership and acculturation, among others.

#### Manager interviews

Managers participated in face-to-face interviews in the stores when customers were not present. The interview obtained information about the manager (e.g., completed at least a high school education or more), their employment and work environment (e.g., years managing store; % of time contact with customers), their employees (e.g., number of full- and part-time employees), their customers (e.g., average number of paying customers per weekday), and the store (e.g., years in operation, square feet, number of SKUs, WIC [Special Supplemental Nutrition Program for Women, Infants, and Children] and SNAP [Supplemental Nutrition Assistance Program] authorization, number of produce distributors, overall sales and produce sales in dollars, and availability of prepared food department).

#### Store audits

Store audits were conducted at baseline to obtain information about the number of cash registers present and working, the number of fixed store aisles, the number and type of different store departments (e.g., prepared food, bakery), availability of a store circular, and number of employees present. These data were used to characterize the stores in the study. Sanchez-Flack et al. [42], reported on the similarities of the intervention and control store environments at baseline, supporting study procedures for randomization to condition.

### Statistical analyses

All analyses were carried out according to the Intent-to-Treat Principle. Intervention effectiveness at 6 months was determined by examining the condition differences at 6 months post-baseline, controlling for baseline values and covariates that were significantly different between conditions. Studies show that adjusting for the baseline values has more power and precision than assessing the change over time using the repeated measure approach [53]. Further, we expected that the intervention effect on our primary outcomes, fruit and vegetable intake, may differ between males and females [54]. Thus, in our models for 6 months, we added gender and the condition-by-gender interaction term in the fixed effect portion of the model to assess for a differential response to the intervention by gender. If the condition-by-gender interaction was not significant, we dropped the interaction term to assess the main effect of condition. We used the linear mixed model to adjust for any store clustering effects (random effect) and customer characteristics (fixed effect). We were powered to detect a difference of 0.3 cups in customers’ reported fruit and vegetable intake at 6 months between intervention and control stores, assuming an ICC of 0.11, recruitment of a minimum of 23 customers per each of 16 stores, and a customer retention rate of 90%, all of which were achieved. Intervention effectiveness (or the extent to which the intervention could continue to show effects in a real-world setting, in this case, with minimal intervention research staff support) at 12 months was determined by examining the condition-by-time (6 to 12 months) interactions, controlling for baseline values and significant covariates between conditions. Similarly, to assess how gender may have moderated intervention effects at 12 months, we added a condition-by-time-by-gender interaction term to the model described previously. If the condition-by-time-by-gender interaction term was not significant, we dropped the three-way interaction term and assessed the condition-by-time interaction term to examine intervention effects at 12 months. Secondary outcomes included fruit and vegetable purchasing and other customer-reported dietary behaviors (i.e., past month variety of fruits; past month variety of vegetables; dietary behavioral strategies to promote fruit and vegetable consumption; percent energy from fat). These models were fitted using SAS PROCMIXED or PROC GLIMMIX.

## Results

### Descriptive customer, manager and store characteristics

Table 1 presents customer information. As shown, with the exceptions of poverty status (*p*=0.004), homeownership (*p*=0.01), and degree of assimilation to the US culture (i.e., non-Hispanic acculturation; *p*=0.003), potential customer level moderators were balanced across conditions. On average, customers were US immigrants in their early 40’s, having immigrated to the US in their twenties. Most were married (72%), with an average household size of five individuals, including two children. Thirty percent identified as male.

**Table 1:** Customer baseline characteristics, overall and by study condition^a^

	Overall *N*=369	Intervention *n*=187	Control *n*=182	
	Mean (SD), Median (Range) or % (n)			*p*-value
**Demographic characteristics**				
Age	42 (12)	41 (11)	43 (13)	0.13
Male	30% (110)	29% (54)	31% (56)	0.69
Foreign born	88% (325)	90% (169)	86% (156)	0.17
Years in the U.S.	19.3 (10.0)	18.3 (9.5)	20.3 (10.4)	0.07
Education				
6 years or less 7-11 years High school degree or more	31% (114) 34% (124) 36% (131)	32% (59) 34% (64) 34% (64)	30% (55) 33% (60) 37% (67)	0.87
Employed	60% (223)	62% (116)	59% (107)	0.52
Married or living as married	72% (256)	78% (143)	65% (113)	0.10
Household size Number of children < 18 yo	5 (1-14) 2 (0-6)	5 (1-14) 2 (0-6)	4 (1-11) 2 (0-6)	0.13 0.19
Homeownership	19.5% (72)	14.4% (27)	24.7% (45)	**0.01**
Living in poverty	72% (256)	78% (143)	65% (113)	**0.004**
Acculturation				
Hispanic^b^ Non-Hispanic^c^	3.5 (0.5) 2.3 (0.8)	3.5 (0.5) 2.2 (0.8)	3.5 (0.5) 2.4 (0.8)	0.97 **0.003**
Receive WIC benefits	33% (120)	35% (66)	30% (54)	0.25
Receive SNAP benefits	33% (120)	35% (66)	30% (54)	0.30
Fruit and vegetable shopping frequency^d^	2.1 (0.6)	2.2 (0.6)	2.1 (0.6)	0.10
**Primary outcomes – daily intake of Fs and Vs**				
Daily cups of fruits	1.4 (1.3)	1.5 (1.5)	1.4 (1.2)	0.84
Daily cups of vegetables	1.1 (0.9)	1.1 (0.9)	1.1 (0.8)	0.69
**Secondary outcomes: Shopping and purchasing**				
Weekly $ spent on groceries	114.8 (57.0)	111.0 (53.4)	118.7 (60.3)	0.19
Weekly $ spent on fruits and vegetables	36.1 (20.4)	34.9 (19.0)	37.3 (22.0)	0.26
Weekly $ spent on groceries at targeted store	41.8 (31.9)	41.0 (30.0)	42.7 (33.9)	0.62
Weekly $ spent on fruits and vegetables at targeted store	16.4 (13.8)	16.5 (13.4)	16.3 (14.1)	0.91
% of weekly $ spent on fruits and vegetables at targeted store from weekly $ spent on groceries at targeted store	41.9 (27.5)	42.1 (28.0)	41.6 (27.0)	0.85
**Secondary outcomes: Variety intake, behavioral strategies and fat intake**				
Past mo. variety of fruits	15.7 (5.7)	15.5 (5.5)	16.0 (5.9)	0.37
Past mo. variety of vegetables	22.1 (6.4)	22.0 (6.4)	22.3 (6.5)	0.63
Beh. Strat. – substitutions^e^	2.4 (0.7)	2.4 (0.7)	2.4 (0.7)	0.32
Beh. Strat. – opting for variety^e^	1.9 (0.6)	1.9 (0.6)	1.8 (0.6)	0.36
Beh. Strat. –preparation^e^	2.4 (0.6)	2.4 (0.6)	2.4 (0.7)	0.47
Percent energy from fat	30.5 (3.4)	30.1 (3.5)	31.0 (3.2)	**0.011**

^a^T-tests were used to examine differences between conditions on continuous variables. Chi-square tests were used to examine differences between conditions on categorical variables

^b^Missing=2; 0.5%

^c^Missing n=3; 0.8%

^d^1=< 1-2/wk, 2=1-2/wk, 3=> 2x/wk

^e^1=never or rarely, 2=sometimes, 3=often, 4=usually

Customers reported consuming 1.4 cups of fruit and 1.1 cups of vegetables daily. Fruit and vegetable shopping occurred 1-2 times/week and customers spent about 42% of their weekly grocery dollars in the targeted stores on fruits and vegetables. The variety of fruits and vegetables consumed were 16 and 22 respectively, and customers reported engaging in healthy dietary behavioral strategies only sometimes [substitution: M=2.4 (0.7), preparation: M=2.4 (0.6); opting for variety: M=1.9 (0.6)]. One condition difference was observed on percent energy from fat; intervention reported consuming less energy from fat (p=0.011) compared to control customers.

Table 2 presents information on the stores and managers. There were no store differences by study condition. Stores had been in business for over 20 years, registered a median of 550 paying customers/day, and averaged approximately 18% of their sales from fresh fruits and vegetables. Most (69%) of the stores were registered as a WIC-authorized store and 88% were authorized SNAP retailers. Finally, using customer-reported fruit and vegetable consumption, the ICC between stores was 0.013 suggesting a minimal degree of clustering of customers within store. Managers were mostly male and one-half were also the store owner. They had managed the store for an average of seven years and a majority (63%) reported receiving retail management training. Given the limited number of full- and part-time employees, 69% of the managers reported spending one-half or more of their day interacting with customers.

**Table 2:** Store and manager baseline characteristics, overall and by study condition^a^

	Overall	Intervention	Control	
	Mean (SD), Median (Range) or % (n)			*p*-value
**Stores**				
Years store in operation^b^	23 (19)	16 (16)	30 (19)	0.13
Sq. footage of sales floor				
Manager/owner report^b^ Measured	5,799 (5,283) 4,083 (3,694)	6,757 (6,473) 3,900 (3,188)	4,840 (4,052) 4,267 (4,360)	0.52 0.85
Number of stock keeping units^b^	5,000 (500-120,000)	4,000 (500-120,000)	7,000 (575-100,000)	0.56
Weekly sales in $^b^	39,250 (27,954)	39,125 (30,053)	39,500 (27,526)	0.98
Weekly produce sales $^b^	7,190 (5,522)	6,971 (6,247)	7,700 (4,424)	0.86
Cash registers	3 (1-5)	3 (1-5)	3 (1-5)	0.75
Aisles	4 (2-9)	4 (3-9)	4 (2-7)	0.79
Avg. customers/weekday^b^	550 (150-1,450)	600 (250-1,450)	450 (150-1,250)	0.53
WIC authorized^b^	69% (11)	63% (5)	75% (6)	0.59
SNAP retailer^b^	88% (14)	100% (8)	75% (6)	0.13
Produce distributors^b^	5.5 (2-15)	5.5 (3-8)	5.5 (2-15)	0.79
Prepared food dept.	38% (6)	38% (3)	38% (3)	1.00
Bakery/Tortilleria	25% (4)	25% (2)	25% (2)	1.00
Number of employees				
Part-time Full-time	3 (0-20) 6 (2-40)	3 (0-8) 5 (3-40)	4 (2-20) 8 (2-30)	0.24 0.75
Store circular available	56% (9)	63% (5)	50% (4)	0.61
**Managers**				
Male	88% (14)	100% (8)	75% (6)	0.13
Latino/Hispanic ethnicity	47% (7)	50% (4)	38% (3)	0.78
Manager was also owner	50% (8)	38% (3)	63% (5)	0.32
High school graduate	88% (14)	88% (7)	88% (7)	1.00
Years managed store	7 (7)	7 (8)	8 (7)	0.67
Training in retail mgmt.	63% (10)	50% (4)	75% (6)	0.30
% of customer contact				0.33
¼ of shift ½ of shift ¾ of shift All day	31% (5) 31% (5) 25% (4) 13% (2)	25% (2) 38% (3) 13% (1) 25% (2)	38% (3) 25% (2) 38% (3) 0% (0)	

^a^T-tests were used to examine differences between conditions on continuous variables. Chi-square tests were used to examine differences between conditions on categorical variables

^b^Based on manager/owner report versus store audit involving observed and measured data

### Retention of customer cohort

As show in Fig. 1, retention rates were 91% at 6 months and 89% at 12 months and similar across the two conditions. Despite the excellent retention rates and no differential loss by condition, some differences were observed between customers who were retained in the study versus those who dropped out at 6 and 12 months. Customers who dropped out versus retained at 6 months were younger (38 versus 43 yrs; *p*=0.023), more likely to be male (50% versus 25%; *p*=0.009), employed (78% versus 59%; *p*=0.032), and live in smaller households (4 versus 5 members; *p*=0.002). Customers who dropped out at 6 months also reported consuming nearly one-half cup more of vegetables and using modification-type behavioral strategies more frequently at baseline compared with those who were retained (daily cups of vegetables: M=1.4, SD=0.9 versus M=1.1, SD=0.8; *p*=0.043; modification strategies: M=2.6, SD=0.6 versus M=2.4, SD=0.6; *p*=0.039). Customers who dropped out versus retained at 12 months were more likely to be male (57% versus 26%; *p*<0.001), report shopping for fruits and vegetables less than once a week (24% versus 10%; *p*=0.028), and spend less money on fruits and vegetables in the targeted stores ($11 versus $17/week for fruits and vegetables; *p*<0.001). (see Additional file 3 for requested information on sample recruitment and analysis).

### Customer changes in dietary intake, purchasing and other dietary behaviors

**Fig. 4:**
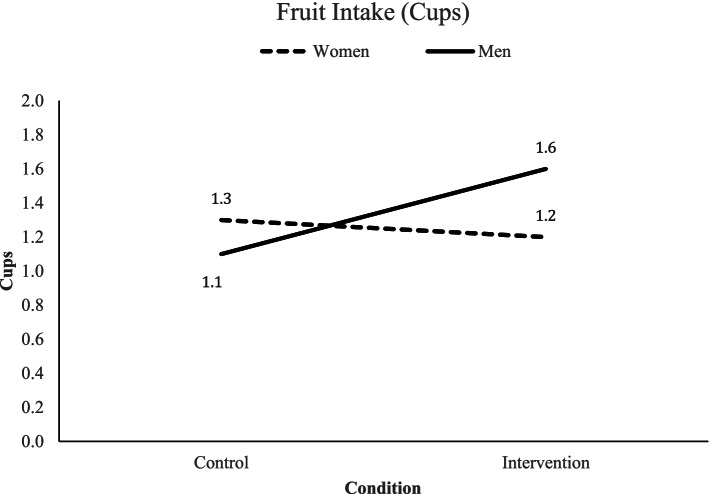
Gender-specific intervention effects at 6 months on daily cups of fruit

Our objectives involved examining for intervention effects on fruit and vegetable consumption, fruit and vegetable purchasing, and other dietary behaviors (past month’s variety of fruits and vegetables, dietary behavioral strategies to promote fruit and vegetable consumption, and percent energy from fat). At 6 months, males in the intervention stores (M=1.6, SD=1.5) consumed more fruit than males in the control stores (M=1.1, SD=0.8), whereas, females in the intervention stores (M=1.2, SD=1.0) consumed less fruit than females in the control stores (M=1.3, SD=1.1; *p*=0.044; see Fig. 4 and Table 3). At 12 months, neither the main effects for condition, time, nor any interaction term (i.e. condition-by-time, condition-by-time-by-gender) were significant for fruit or vegetable consumption. What appeared to happen was a drop in fruit consumption among males in the control condition at 6 months that was not observed among males in the intervention condition.

**Table 3:** Raw means for customers’ outcomes at 6 and 12 months by gender, condition, and their interaction

Outcome	Time		Control		Intervention		6 months^a^		12 months^b^	
			Women Mean (SD)	Men Mean (SD)	Women Mean (SD)	Men Mean (SD)	Condition main effect *p*-value	Condition × Gender *p*-value	Condition × Time *p*-value	Condition × Time × Gender *p*-value
**Primary outcomes: Daily intake of fruits and vegetables**										
Daily cups of fruit	Baseline	(*N*=369)	1.4 (1.2)	1.4 (1.2)	1.4 (1.5)	1.6 (1.5)	0.979	**0.044**	0.493	0.597
	6-mos	(*N*=337)	1.3 (1.1)	1.1 (0.8)	1.2 (1.0)	1.6 (1.5)				
	12-mos	(*N*=327)	1.2 (1.2)	1.4 (1.2)	1.2 (1.2)	1.7 (1.3)				
Daily cups of vegetables	Baseline	(*N*=368)	1.1 (0.8)	1.0 (0.8)	1.1 (0.8)	1.1 (1.0)	0.602	0.368	0.871	0.900
	6-mos	(*N*=336)	1.1 (0.6)	0.9 (1.0)	1.0 (0.8)	1.1 (0.9)				
	12-mos	(*N*=327)	1.1 (0.8)	0.9 (0.5)	1.0 (0.6)	1.1 (0.7)				
**Secondary outcomes: Shopping and purchasing**										
Weekly $ spend on groceries overall	Baseline	(*N*=368)	122.4 (61.7)	110.4 (56.7)	113.2 (53.0)	105.4 (54.6)	0.637	0.270	0.172	0.896
	6-mos	(*N*=334)	114.0 (49.6)	106.0 (60.3)	109.1 (52.2)	118.9 (69.5)				
	12-mos	(*N*=326)	117.7 (55.8)	115.6 (56.0)	108.3 (48.9)	115.4 (59.0)				
Weekly $ spent on fruits and vegetables overall	Baseline	(*N*=367)	39.2 (23.1)	33.2 (18.6)	35.9 (19.6)	32.7 (16.6)	0.643	0.393	0.672	0. 405
	6-mos	(*N*=332)	38.7 (21.2)	33.8 (17.3)	39.2 (20.0)	32.1 (15.7)				
	12-mos	(*N*=326)	39.7 (24.2)	34.9 (20.4)	39.9 (20.4)	37.0 (21.1)				
Weekly $ spent on groceries at targeted store	Baseline	(*N*=367)	45.7 (36.9)	35.7 (24.6)	31.9 (32.4)	38.9 (22.4)	0.474	**0.026**	0.129	0.071
	6-mos	(*N*=330)	43.5 (32.8)	27.0 (15.6)	46.4 (31.9)	47.9 (42.6)				
	12-mos	(*N*=325)	43.2 (44.7)	31.4 (25.9)	45.4 (34.6)	37.9 (33.4)				
Weekly $ spent on fruits and vegetables at targeted store	Baseline	(*N*=366)	18.1 (15.2)	12.4 (10.3)	17.5 (14.6)	13.9 (9.6)	0.785	0.358	0.786	0.476
	6-mos	(*N*=333)	17.8 (14.9)	10.7 (9.0)	19.6 (15.1)	16.1 (13.8)				
	12-mos	(*N*=325)	17.1 (20.0)	12.3 (11.1)	20.1 (17.1)	14.2 (11.7)				
% of weekly $ spent on fruits and vegetables at targeted store from weekly $ spent on groceries at targeted store	Baseline	(*N*=362)	43.4 (28.0)	37.3 (24.5)	43.8 (29.0)	38.0 (25.2)	0.580	0.566	0.091	0.994
	6-mos	(*N*=312)	45.0 (26.5)	44.1 (32.5)	43.8 (28.5)	38.8 (21.7)				
	12-mos	(*N*=289)	42.8 (25.6)	40.5 (30.9)	48.1 (30.8)	41.4 (22.6)				
**Secondary outcomes: Variety intake, behavioral strategies and fat intake**										
Past month variety of fruits	Baseline	(*N*=336)	16.2 (5.7)	15.5 (6.2)	16.0 (5.3)	14.1 (5.7)	0.883	0.677	0.701	0.188
	6-mos	(*N*=337)	16.7 (5.2)	16.3 (5.8)	16.2 (5.1)	15.5 (5.8)				
	12-mos	(*N*=327)	17.0 (5.3)	14.9 (5.7)	16.3 (5.4)	16.2 (4.8)				
Past month variety of vegetables	Baseline	(*N*=369)	22.6 (6.1)	21.6 (7.3)	22.5 (6.3)	20.8 (6.4)	0.519	0.102	0.200	0.942
	6-mos	(*N*=337)	23.5 (6.2)	21.6 (6.3)	22.4 (5.8)	22.2 (6.2)				
	12-mos	(*N*=327)	22.8 (5.7)	21.0 (7.0)	22.7 (6.9)	23.0 (5.4)				
Behavioral Strategies – substitutions	Baseline	(*N*=369)	2.5 (0.7)	2.2 (0.6)	2.5 (0.6)	2.2 (0.8)	0.561	0.453	0.972	0.381
	6-mos	(*N*=336)	2.6 (0.6)	2.3 (0.6)	2.6 (0.6)	2.4 (0.6)				
	12-mos	(*N*=327)	2.6 (0.6)	2.4 (0.6)	2.5 (0.6)	2.5 (0.7)				
Behavioral strategies – opting for variety	Baseline	(*N*=342)	1.9 (0.6)	1.6 (0.5)	2.0 (0.6)	1.7 (0.6)	0.728	0.330	0.110	0.379
	6-mos	(*N*=326)	2.0 (0.7)	1.8 (0.6)	2.0 (0.6)	1.9 (0.6)				
	12-mos	(*N*=318)	2.0 (0.6)	1.7 (0.6)	2.0 (0.6)	1.9 (0.7)				
Behavioral strategies – preparation	Baseline	(*N*=362)	2.5 (0.7)	2.1 (0.6)	2.4 (0.5)	2.2 (0.6)	0.736	0.937	**0.013**	0.929
	6-mos	(*N*=335)	2.6 (0.6)	2.2 (0.6)	2.5 (0.6)	2.3 (0.6)				
	12-mos	(*N*=326)	2.5 (0.6)	2.1 (0.6)	2.5 (0.5)	2.3 (0.6)				
Percent energy from fat	Baseline	(*N*=355)	30.7 (3.4)	31.5 (2.6)	29.5 (3.4)	31.6 (3.2)	0.741	0.764	0.117	0.820
	6-mos	(*N*=327)	30.4 (3.3)	31.6 (2.4)	30.2 (3.1)	31.2 (3.1)				
	12-mos	(*N*=315)	30.9 (3.4)	31.5 (2.6)	29.9 (3.2)	31.2 (3.2)				

^a^Baseline to 6-months tested for intervention effects immediately post-intervention delivery supported by the research team. Model was adjusted for non-Hispanic acculturation score, poverty, and homeownership given baseline condition differences

^b^6-months to 12-months tested for intervention effects immediately after the store maintenance phase. Model was adjusted for non-Hispanic acculturation score, poverty, and homeownership given baseline condition differences

In terms of purchasing, a condition-by-gender interaction was observed at 6 months on weekly dollars spent on overall groceries at the targeted store. Males spent more money on groceries in the targeted intervention stores (M=47.9, SD=42.6) than males in the targeted control stores (M=27.0, SD=15.6) in a typical week (*p*=0.026). The same pattern was observed for females (Intervention: M=46.4, SD=31.9; Control: M=43.5, SD=32.8) although differences were substantially greater for males than females. However, this did not translate to differences in % of weekly dollars spent on fruits and vegetables from among all grocery dollars spent at the targeted store at 6 months. In addition, although not significant, it appears that males in the intervention versus control condition reduced their weekly dollars spent on overall groceries in the targeted store at 12 months, at the end of Phase 3 of the intervention; this same pattern was not observed among females (12-month condition-by-time-by-gender interaction, *p*=0.071). There were no other significant main effects for condition, time, nor any interaction term (i.e. condition-by-time, condition-by-time-by-gender) at 12 months.

In terms of other outcomes, the only intervention effect observed was on preparation-related behavioral strategies at 12 months, with evidence showing significantly more frequent engagement in preparation behaviors among intervention versus control condition customers (*p*=0.013). There were no intervention effects on fruit and vegetable variety, two of three dietary behavioral strategies, or percent energy from fat at 6 or 12 months and no interactions with gender. Importantly, there were no baseline condition differences in shopping frequency and no changes in shopping frequency by condition at 6 and 12 months.

## Discussion

An intervention designed to increase the purchasing and consumption of fruits and vegetables among Latino/Hispanic store customers was effective at improving consumption of fruit at 6 months among males versus females by preventing a drop observed among male customers in the control condition. However, these same findings were not observed at 12 months. These findings are notable as they suggest that an environmental change intervention may be moderately effective at modifying diet in the short-term. However, health behavior change may require ongoing environmental support as evidenced by an increase in dollars spent on groceries in the targeted stores among males in the intervention condition at 6 months followed by a drop at 12 months. Consistent with our conceptual model and intervention approach [24], structural environmental changes may be key for long-term health behavior change.

Regarding the effects on males, there is evidence to support community-based interventions improving health outcomes among men but not women [55], and specifically interventions that involve environmental changes to improve diet [56]. In addition, there is some evidence to suggest that interventions that do not require active engagement around dietary change may not threaten masculine norms about diet and eating [57]. Likewise, the cross-product marketing that occurred within the butcher section may have helped to reach men selecting meat to purchase. Also, convenience was identified as an important factor to optimize the change process among Latino men to promote weight management [58]. Furthermore, the finding that male customers in the intervention condition reported spending more money on groceries at the targeted store compared with male customers in the control condition at 6 months is advantageous to the stores as they try and retain a share of the market in an increasingly competitive food environment. Men are now recognized as important contributors to household food purchases [59], and they are important to consider in future food purchasing research.

### Strengths and limitations

Study strengths included the controlled study design, the excellent retention rates, characteristics of the study sample, the focus on US Latino/Hispanic stores, and building on an effectively-delivered pilot study. Regarding the study design, this is among the few studies to describe the results of a store-based C-RCT on self-reported behavior changes in a cohort of customers over 12 months [19, 60, 61]. Regarding the retention rates, our customer retention rates were exceptional and maximized through a rigorous recruitment, selection and retention process. Few customers were lost-to-follow-up despite having been recruited while walking into a limited assortment food store. There were few, although important, differences between those who dropped out versus retained (younger, male, healthier dietary behaviors although shopped less frequently and spent less money on fruits and vegetables). In terms of the sample of customers, this study is unique in having recruited males to participate on the evaluation cohort. Increasingly, researchers and practitioners are recognizing the important role that men play in determining their own dietary intake and the intake of family members [62–64]. Finally, we controlled for the few customer-related factors that were different between the intervention and control conditions at baseline.

In terms of store-related strengths, although regional differences in the management of limited assortment food stores have been observed [65–69], the selection and screening processes of stores strengthens the generalizability of study findings to other limited assortment food stores in the US that cater to a largely Latino/Hispanic customer base. Substantial challenges were experienced recruiting stores, including multiple face-to-face visits with managers. Thus, our ability to retain all stores was remarkable. An additional strength of this study is its focus on ethnic-focused food stores catering to the Latino/Hispanic population, the second fastest growing ethnic group in the US. Latino/Hispanic-focused food stores and the fruit and vegetable purchasing and consumption behaviors of Latino/Hispanic adults have not been well represented in the in-store intervention literature. Understanding how to influence purchasing behavior within this food environment that seeks to reach Latino/Hispanic customers is critical for reducing obesity and other health disparities.

The primary limitation of the study was the reliance on self-report for all outcomes. Our original grant proposal included the collection of biospecimens to examine changes in serum carotenoids, as a biomarker of fruit and vegetable intake, in a subgroup of customers; however, funding cuts resulted in the loss of this protocol. In addition, we intended to obtain store sales data to quantify purchases. Unfortunately, most of the stores’ point-of-sale systems either did not provide itemized receipts or only had crude indicators for product categories, as experienced in previous research with similar types of stores [18, 66]. Further, some store managers refused to provide sales data given previous negative experiences with researchers and others. Thus, although error was consistent across study conditions, the validity of the purchasing measures remain a question [17].

Related to the primary outcomes, it is possible that limiting the sample recruited to those who consumed four or fewer cups of fruits and vegetables every day reduced generalizability of study findings. This was done to minimize the potential for a ceiling effect; the criterion of four cups daily was selected to reflect current fruit and vegetable consumption patterns among US Latinos/Hispanics. Finally, although the intervention was implemented as intended during Phases 1 and 2, differences in measures of availability and placement of fruits and vegetables between intervention and control stores at the end of Phase 2 were not observed [42], thus limiting our ability to conclude what changes may have influenced purchases.

### Future directions

The findings regarding an increase in dollars spent on groceries from the targeted stores require further exploration, in part to determine how purchasing is related to diet quality. There is evidence to indicate that store-based interventions can promote the purchasing of healthy products [18, 19, 22, 61, 70]. More research is needed on how to maintain these types of changes in customers’ purchasing behaviors, and the extent to which they reflect actual consumption.

The findings regarding gender differences in intervention effects on fruit consumption require further exploration. There is evidence to suggest that males may be more promotionally sensitive than females [71]. There is also evidence to suggest that males versus females may be more receptive and engaged with interventions that are more passive in nature and that do not require active involvement in the change process. To test these hypotheses, future research could be designed to examine whether point-of-purchase strategies work better for males versus females. Likewise, there is evidence to indicate that fruit consumption may be easier to change than vegetable consumption generally and within a store-based intervention. Fruits are more appealing than vegetables and they may be easier to promote in forms that are appealing to customers (e.g., convenience to eat).

## Conclusions

This is among a small set of real-world studies in food stores [70] to demonstrate the effectiveness of a store-based intervention to change customers’ self-reported grcoery purchasing and male customers’ consumption of fruit in the short-term. Identifying strategies that lead to longer-term changes among customers are needed.

## Supplementary Information

**Additional file 1.** CONSORT checklist for clustered randomized controlled trial (C-RCT). Consolidated Standards of Reporting Trials checklist shows the evidence-based, minimum set of recommendations for reporting randomized trials. The CONSORT checklist includes 25 items, and is accompanied by the CONSORT flow diagram (Fig. 1).

**Additional file 2.** TIDieR checklist. The Template for Intervention Description and Replication is a 12-item checklist providing information to describe an intervention, and the location of the information within the manuscript.

**Additional file 3.** Empirical study requested additional information. Data on how the sample was recruited, how representative the sample was of the target group, how the analysed sample differed from the recruited sample and how any missing data were handled.

**Additional file 4: Supplement Table 1.** The intervention effect and adjusted means for customers’ outcomes at 6- and 12-months post-baseline by gender and condition.

## Data Availability

The datasets used and/or analyzed during the current study are available from the corresponding author on reasonable request.

## Abbreviations

CONSORT: Consolidated Standards of Reporting Trials
C-RCT: Clustered randomized controlled trial
SKU: Stock keeping unit
TIDieR: Template for Intervention Description and Replication
USA: United States of America
