# Supplementary material for: The El Valor de Nuestra Salud clustered randomized controlled trial store-based intervention to promote fruit and vegetable purchasing and consumption
Source: Int J Behav Nutr Phys Act. 2022 Feb 17;19:19. doi: 10.1186/s12966-021-01220-w (PMC8851758; doi:10.1186/s12966-021-01220-w)
Supplement: Supplementary file 3 — Additional file 3. Empirical study requested additional information. Data on how the sample was recruited, how representative the sample was of the target group, how the analysed sample differed from the recruited sample and how any missing data were handled. [file 12966_2021_1220_MOESM3_ESM.docx]

**Empirical study: requested additional information**

Please find below the additional information requested for all empirical studies.

1. **How the sample was recruited***

Tienda enumeration and recruitment

A systematic enumeration of all food stores in San Diego County was conducted using five sources: county food permits, county health department registry, the Special Supplemental Nutrition Program for Women, Infants, and Children (WIC) program, the Supplemental Nutrition Assistance Program (SNAP), and a previous observational study conducted in the target area. Duplicate entries were removed resulting in a final list of 5601 outlets eligible for the second phase of screening which involved eliminating those clearly identifiable as other than a food store (e.g., car wash, electronics store, nonretail; n=1488). This new list of 4113 outlets was further reduced by excluding zip codes where US 2000 Census data indicated that the proportion of Hispanic/Latino residents was less than 20% (given our focus on Latinos), as well as San Diego’s South County given competing intervention activities in stores led primarily by the county health department (n=2437). The remaining 1676 outlets were then filtered to exclude known super-centers, supermarkets, liquor stores, convenience stores, dollar stores, restaurants, etc. and additional duplicates were removed (n=1110). This left 566 that was further reduced to 339 outlets after excluding those ineligible based on internet or phone verification.

Because 2010 Census data were not available when store enumeration was first conducted, 2000 Census data were used to identify zip codes with sufficient Latino representation. Over time demographic shifts occurred that changed the ethnic composition of various neighborhoods. During the final round of tienda recruitment, given time and resource constraints for traveling to more distant stores, the team identified four additional zip codes in proximity to the study offices that contained census tracts representing at least 20% Latino population. An additional 365 outlets were identified in those zip codes (out of the original 5601). Of those, 268 were dropped because they were identified as an outlet other than a grocery store, then another 54 were excluded because they did not fall within census tracts that contained at least 20% Latino population, leaving an additional 43 outlets to be verified. This brought the total available for verification and potential recruitment to 382.

In-person verification involved a store audit to determine whether the outlet was a food store, and if yes, whether it met study inclusion criteria: carried some fresh produce; had a serviced meat department; was considered small or medium-to-large based on the number of cash registers (i.e., present and working) and fixed store aisles (for matching purposes); and catered to a Latino clientele (e.g., had Spanish language signage; some or all employees observed speaking Spanish; some products from Mexico and other Latin-American countries including specific cuts of meat in the serviced meat department, specific dishes in the serviced prepared food department if available). No criteria were used for number of employees present given the wide variation observed in previous studies with similar stores.

Of this 382 outlets, 273 (71%) were not eligible for various reasons from being a convenience store to no longer in business. Six (1.5%) additional duplicates were found and during ground truthing, 26 new stores were found and added, leaving 129 in the recruitment pool. From among these, 84 tiendas were approached for participation. To minimize the potential for cross-contamination, the 84 tiendas were sorted geographically; once one tienda was recruited, possible pair-matched tiendas were identified that were located at least one mile away from the recruited tienda.

Initial tienda recruitment strategies involved mailing an introductory letter followed by an in-store visit or a phone call to schedule a meeting. However, after several attempts, it was determined that many tienda managers did not recall receiving the letter. Thus, the approach was modified to include a drop-in visit with a fact sheet and introductory letter in-hand, recruitment on-the-spot, or scheduling a meeting to introduce the study to the tienda manager at a later date. The fact sheet included a pilot-study tienda manager testimonial and a testimonial by the president of a regional trade association for independent retailers along with answers to frequently asked questions. During the recruitment meeting with the tienda manager, the project staff member reviewed study requirements and gave the tienda manager a packet to review. If the tienda manager was interested, the staff member completed an initial eligibility screener which involved confirming that the tienda was eligible. If eligibility was met and the manager agreed, the two parties would review and sign a memorandum of understanding.

Manager and Employee recruitment

Tienda manager eligibility criteria included being at least 18 years of age; having worked at least 20 hours per week for the participating tienda for a minimum of six months; planning to continue as tienda manager for the next 12 months; having had or having decision-making authority in the tienda; being willing to consider providing sales data to researchers; and not being employed at another participating tienda. If the tienda manager was eligible, the project staff member conducted the baseline assessment with him/her. Recruitment of employees to participate in evaluation activities was dropped due to funding cuts. As such, employee recruitment was limited to intervention involvement and their involvement in the intervention is described in that section.

Primary study participants: Tienda customers recruitment

To screen for eligibility, customers were approached by trained bilingual (English/Spanish) research assistants as they were entering the *tienda*. As soon as a customer agreed to be screened, recruitment of other customers stopped until all activities were completed with that individual. If the customer expressed initial interest, the research assistant screened him/her for the following eligibility criteria: at least 18 years of age; identified as Latino/Hispanic; primary food shopper for his/her household; purchased food products at the participating *tienda* at least once per week; not presently consuming more than four cups of FV per day; not having dietary restrictions limiting FV consumption; fluency in English and/or Spanish and able to read Spanish; not shopping at another participating *El Valor tienda* once a month or more; having a telephone; planning to remain in San Diego County for the next 12 months; and not participating in another research study. To ensure independent observations, only one individual per household could participate. If the customer was eligible and agreed to participate, the research assistant collected the baseline data immediately or scheduled a future visit to collect these data. If the customer refused at any point during recruitment, the refusal was noted, along with important demographic factors such as gender to characterize those who refused. If the customer was ineligible, he/she was thanked for his/her time and ineligibility criteria were noted along with similar demographic factors as those who refused.

1. **How representative the sample was of the target group***

We were successful recruiting customers who visited these stores at least once a week. Thus, they are regular customers. Comparing the demographics of the recruited sample to the target population within these communities using U.S. Census data, our sample is fairly representative of the San Diego Latino/Hispanic population on dimensions such as marital status and household size. It is not expected that the sample was more female than male given gender roles in food purchasing.

1. **How the analysed sample differed from the recruited sample**

We provide extensive information in the manuscript regarding how those who dropped out differed from those who were recruited to participate at baseline (see Lines 645-659).

1. **How any missing data were handled**

All analyses were carried out according to the Intent-to-Treat Principle consistent with standard practice in most trials.

* For more information, please refer to Ayala GX, Baquero B, Pickrel JL, et al. A store-based intervention to increase fruit and vegetable consumption: The El Valor de Nuestra Salud cluster randomized controlled trial. Contemp Clin Trials. 2015;42:228-238.
