# Supplementary material for: The El Valor de Nuestra Salud clustered randomized controlled trial store-based intervention to promote fruit and vegetable purchasing and consumption
Source: Int J Behav Nutr Phys Act. 2022 Feb 17;19:19. doi: 10.1186/s12966-021-01220-w (PMC8851758; doi:10.1186/s12966-021-01220-w)
Supplement: Supplementary file 4 — Additional file 4: Supplement Table 1. The intervention effect and adjusted means for customers’ outcomes at 6- and 12-months post-baseline by gender and condition. [file 12966_2021_1220_MOESM4_ESM.docx]

Supplement Table 1. The intervention effect and adjusted means for customers’ outcomes at 6- and 12-months post-baseline by gender and condition.

| **Outcome** | **Time** | | **Control** | |  | **Intervention** | |  | **6 months^1^** | |  | **12 months^2^** | |
| --- | --- | --- | --- | --- | --- | --- | --- | --- | --- | --- | --- | --- | --- |
|  |  |  | **Women**  Adjusted mean (SE) | **Men**  Adjusted mean (SE) |  | **Women**  Adjusted mean (SE) | **Men**  Adjusted mean (SE) |  | **Condition Main Effect**  p-value | **Condition ×**  **Gender**  p-value |  | **Condition**  **×**  **Time**  p-value | **Condition × Time × Gender**  p-value |
| **Primary outcomes: Daily intake of Fs and Vs** | | | | | | | | | | | | | |
| Daily cups of fruits | 6-mos | (N=337) | 1.3  (0.12) | 1.1  (0.17) |  | 1.2  (0.12) | 1.5  (0.17) |  | 0.979 | **0.044** |  | - | - |
|  | 12-mos | (N=327) | 1.2  (0.10) | 1.3  (0.17) |  | 1.3  (0.10) | 1.7  (0.17) |  | - | - |  | 0.493 | 0.597 |
| Daily cups of vegetables | 6-mos | (N=336) | 1.1  (0.07) | 1.1  (0.11) |  | 1.1  (0.08) | 1.2  (0.12) |  | 0.602 | 0.368 |  | - | - |
|  | 12-mos | (N=327) | 1.1  (0.07) | 0.9  (0.11) |  | 1.0  (0.07) | 1.2  (0.11) |  | - | - |  | 0.871 | 0.900 |
| **Secondary outcomes: Shopping and purchasing** | | | | | | | | | | | | | |
| Weekly $ spend on groceries overall | 6-mos | (N=334) | 111.1 (4.78) | 115.5 (7.11) |  | 111.9 (5.04) | 129.6 (7.33) |  | 0.637 | 0.270 |  | - | - |
|  | 12-mos | (N=326) | 114.2 (4.18) | 119.1 (6.85) |  | 109.3 (4.13) | 122.8 (6.89) |  | - | - |  | 0.172 | 0.896 |
| Weekly $ spent on fruits and vegetables overall | 6-mos | (N=332) | 37.0 (1.95) | 35.8 (2.80) |  | 38.7 (2.06) | 33.6 (2.87) |  | 0.643 | 0.393 |  | - | - |
|  | 12-mos | (N=326) | 38.5 (1.70) | 36.5 (2.78) |  | 40.5 (1.68) | 39.3 (2.79) |  | - | - |  | 0.672 | 0. 405 |
| Weekly $ spent on groceries at targeted store | 6-mos | (N=330) | 41.2 (2.64) | 31.8 (4.06) |  | 45.2 (2.80) | 52.4 (4.12) |  | 0.475 | **0.026** |  | - | - |
|  | 12-mos | (N=325) | 40.9 (2.61) | 37.6 (4.32) |  | 44.8 (2.57) | 40.6 (4.28) |  | - | - |  | 0.129 | 0.071 |

| **Outcome** | **Time** | | **Control** | |  | **Intervention** | |  | **6 months^1^** | |  | **12 months^2^** | |
| --- | --- | --- | --- | --- | --- | --- | --- | --- | --- | --- | --- | --- | --- |
|  |  |  | **Women**  Adjusted mean (SE) | **Men**  Adjusted mean (SE) |  | **Women**  Adjusted mean (SE) | **Men**  Adjusted mean (SE) |  | **Condition Main Effect**  p-value | **Condition ×**  **Gender**  p-value |  | **Condition**  **×**  **Time**  p-value | **Condition × Time × Gender**  p-value |
| Weekly $ spent on fruits and vegetables at targeted store | 6-mos | (N=333) | 17.3 (1.45) | 12.7 (2.03) |  | 18.9 (1.52) | 17.3 (2.10) |  | 0.785 | 0.358 |  | - | - |
|  | 12-mos | (N=325) | 16.2 (1.29) | 14.5 (2.12) |  | 19.8 (1.26) | 16.1 (2.15) |  | - | - |  | 0.786 | 0.476 |
| % of weekly $ spent on fruits and vegetables at targeted store from weekly $ spent on groceries at targeted store | 6-mos | (N=312) | 45.3 (2.63) | 43.3 (4.29) |  | 44.6 (2.53) | 38.6 (4.16) |  | 0.580 | 0.566 |  | - | - |
|  | 12-mos | (N=289) | 42.6 (2.73) | 41.0 (4.50) |  | 48.3 (2.56) | 42.8 (4.72) |  | - | - |  | 0.091 | 0.994 |
| **Secondary outcomes: Variety intake, behavioral strategies and fat intake** | | | | | | | | | | | | | |
| Past month variety of fruits | 6-mos | (N=337) | 16.4 (0.57) | 15.8 (0.78) |  | 16.3 (0.59) | 16.3 (0.79) |  | 0.883 | 0.677 |  | - | - |
|  | 12-mos | (N=327) | 16.7 (0.43) | 14.5 (0.71) |  | 16.4 (0.42) | 17.0 (0.71) |  | - | - |  | 0.701 | 0.188 |
| Past month variety of vegetables | 6-mos | (N=337) | 23.2 (0.54) | 21.6 (0.80) |  | 22.2 (0.57) | 22.9 (0.82) |  | 0.519 | 0.102 |  | - | - |
|  | 12-mos | (N=327) | 22.6 (0.50) | 21.3 (0.82) |  | 22.7 (0.49) | 23.5 (0.82) |  | - | - |  | 0.200 | 0.942 |
| Behavioral Strategies – substitutions | 6-mos | (N=336) | 2.6  (0.04) | 2.5  (0.07) |  | 2.6  (0.04) | 2.6  (0.07) |  | 0.561 | 0.453 |  | - | - |
|  | 12-mos | (N=327) | 2.5  (0.05) | 2.4  (0.08) |  | 2.5  (0.05) | 2.6  (0.08) |  | - | - |  | 0.972 | 0.381 |

| **Outcome** | **Time** | | **Control** | |  | **Intervention** | |  | **6 months^1^** | |  | **12 months^2^** | |
| --- | --- | --- | --- | --- | --- | --- | --- | --- | --- | --- | --- | --- | --- |
|  |  |  | **Women**  Adjusted mean (SE) | **Men**  Adjusted mean (SE) |  | **Women**  Adjusted mean (SE) | **Men**  Adjusted mean (SE) |  | **Condition Main Effect**  p-value | **Condition ×**  **Gender**  p-value |  | **Condition**  **×**  **Time**  p-value | **Condition × Time × Gender**  p-value |
| Behavioral strategies – opting for variety | 6-mos | (N=326) | 2.0  (0.05) | 1.9  (0.09) |  | 2.0  (0.06) | 2.0  (0.09) |  | 0.728 | 0.330 |  | - | - |
|  | 12-mos | (N=318) | 1.9  (0.06) | 1.8  (0.10) |  | 2.0  (0.06) | 1.9  (0.09) |  | - | - |  | 0.110 | 0.379 |
| Behavioral strategies – preparation | 6-mos | (N=335) | 2.6  (0.04) | 2.4  (0.08) |  | 2.5  (0.05) | 2.4  (0.08) |  | 0.736 | 0.937 |  | - | - |
|  | 12-mos | (N=326) | 2.4  (0.05) | 2.3  (0.08) |  | 2.6  (0.05) | 2.4  (0.08) |  | - | - |  | **0.013** | 0.929 |
| Percent energy from fat | 6-mos | (N=327) | 30.2 (0.39) | 31.2 (0.54) |  | 30.3 (0.40) | 31.1 (0.53) |  | 0.741 | 0.764 |  | - | - |
|  | 12-mos | (N=315) | 30.8 (0.28) | 31.2 (0.49) |  | 30.2 (0.28) | 30.9 (0.46) |  | - | - |  | 0.117 | 0.820 |

^1^Changes between baseline and 6-months tested for intervention effects immediately post-intervention delivery supported by the research team. Model was adjusted for non-Hispanic acculturation score, poverty, and homeownership given baseline condition differences.

^2^Changes between 6-months and 12-months tested for intervention effects immediately after the store maintenance phase. Model was adjusted for non-Hispanic acculturation score, poverty, and homeownership given baseline condition differences.
